# A Principle of Intentionality

**DOI:** 10.3389/fpsyg.2017.00137

**Published:** 2017-02-07

**Authors:** Charles K. Turner

**Affiliations:** Retired, BrentwoodCA, USA

**Keywords:** principle of intentionality, intentional relation, intention, mental processes, principle of causality

## Abstract

The mainstream theories and models of the physical sciences, including neuroscience, are all consistent with the principle of causality. Wholly causal explanations make sense of how things go, but are inherently value-neutral, providing no objective basis for true beliefs being better than false beliefs, nor for it being better to intend wisely than foolishly. Dennett (1987) makes a related point in calling the brain a syntactic (procedure-based) engine. He says that you cannot get to a semantic (meaning-based) engine from there. He suggests that folk psychology revolves around an intentional stance that is independent of the causal theories of the brain, and accounts for constructs such as meanings, agency, true belief, and wise desire. Dennett proposes that the intentional stance is so powerful that it can be developed into a valid intentional theory. This article expands Dennett’s model into a principle of intentionality that revolves around the construct of objective wisdom. This principle provides a structure that can account for all mental processes, and for the scientific understanding of objective value. It is suggested that science can develop a far more complete worldview with a combination of the principles of causality and intentionality than would be possible with scientific theories that are consistent with the principle of causality alone.

## Introduction

One powerful approach to theorizing about how things go in the world is by model-building. A model is a representation of something real, and includes hypothetical entities such as influences, constructs, and relations. The model predicts how things will go in some aspect of reality, and provides one explanation of it. There is not necessarily any assertion that its hypothetical entities mirror real entities. Model-building in the area of folk psychology is discussed in Maibom (2003) and Godfrey-Smith (2005).

Any scientific explanation of change is likely to invoke non-physical entities such as forces and causal relations. Although they might *seem* manifest, they are in some sense inferred, and thus hypothetical. In that sense, virtually all scientific explanation of change occurs in the form of models. Prediction, too, is largely model-based, although scientific observation such as Tycho Brahe’s records of astronomical movement allows prediction in the absence of any model.

In some scientific explanation, hypothetical entities are believed to mirror actual entities. A particularly obvious example is that space-time is non-physical, but is taken to be an aspect of reality. Still, there is value in the notion that even space-time is a hypothetical entity, subject to being modified or replaced as understanding grows. Famously, Einstein transformed scientific beliefs about space and time. And Kant suggested that space and time were simply *a priori* categories of the understanding, rather than aspects of ultimate (noumenal) reality. Even when there is substantial reason to believe that a certain hypothetical structure precisely mirrors how things actually are (as with the relation *E* = *mc*^2^), hypothetical structures are invented models^1^, and the evidence that confirms their power in prediction and explanation draws broadly from other, often implicit hypothetical entities, such as the principle of causality.

The science of psychology can especially benefit by treating influences on change as mere hypothetical entities that are model-dependent. The mind is intuitively modeled as an intentional system, whereas the brain is modeled as a causal system. These might both be valid models, even if intentionality is inconsistent with the principle of causality. And allowing intentional models to stand on their own might open the door to there being various human sciences that revolve around models that are inconsistent with the wholly causal models of the physical sciences.

All mainstream scientific models seem to be causal models, treating any consistencies in physical events as somehow conforming to the principle of causality.^2^ Roughly, there is a causal relation wherever, apart from randomness, physical event B always immediately follows a spatially and temporally contiguous physical event A, such that event B will not occur if event A is blocked. The principle of causality asserts that, apart from randomness, every physical event can be traced to one or more causes, and thus through causal chains into the past (quickly muddied by randomness). There is enormous value in finding ways to model all physical change as consistent with the principle of causality. For example, when quantum events turned out not to follow the principle of causality, a small adaptation of the principle solved the problem. By treating event A as a large number of repetitions of a certain cause, the reliable effect is a fixed statistical distribution that can be treated as event B. Said differently, each single event A causes a certain wave function as event B. Quantum physics conflicts slightly with the principle of causality in other ways. Bell’s theorem describes causal relationships that violate the requirement of contiguity, and there are theoretical approaches in which a quantum effect occurs slightly prior to its cause. As with any hypothetical entity, the principle of causality is subject to modification with new evidence of these sorts.^3^

Presumably, the principle of causality, in some form, will turn out to hold universally for physical events. This article proposes a principle of intentionality that is inconsistent with the principle of causality. It is expected to hold universally for all voluntary behavior, even if all mental processes are consistent with brain processes, and all brain processes are consistent with the principle of causality. That is, an intentional model of the mind and a causal model of the brain might both be valid, built around hypothetical structures that are inconsistent with each other, and might or might not mirror the structure of reality.^4^

According to the hypothesis developed below, the principle of intentionality not only guides all voluntary thought and behavior, but is also implicated in all meaning, value, and purpose. If it turns out to be valid, this will empower models of the mind that might be far more powerful than any wholly causal model of mental processes can be. Further, it might offer powerful models of objective value and purpose, with implications in other human sciences beyond psychology.

There is reason to doubt that causal models can adequately account for agency or objective value. Causal models show how things *will* go, but not how intentions can change the course of physical events, nor why one direction is better than another. They show what *is* true, but not how true beliefs are any more objectively valuable than false beliefs. They seem to be What-Is models, passively describing the universe as if it were value-neutral. The principle of intentionality is developed below in a manner that opens the door to value, such that one thing is objectively better than another.

If the principle of intentionality enables powerful modeling of both minds and value (how things matter objectively), this might result in a Copernican revolution, in which the principle of causality is no longer the center of the scientific universe (in which valid scientific theories can be inconsistent with that principle). Instead, beliefs about the world as a whole might come to revolve around What-Matters models, in which minds and mattering are scientifically validated, with What-Is models as subsets that are employed to make sense of only the physical aspects of the universe. A What-Matters model would employ a combination of the principles of intentionality and causality, making sense of some of the key constructs that dominate human life, as to minds (such as consciousness, agency, beliefs, and desires), and mattering (such as truth, good, beauty, and purpose).

The focus of this article will be a bit more modest: an intentional model for predicting and explaining mental processes within the science of psychology, descriptive of minds but not prescriptive of behavior. It will become clear how such a model might someday lead to significant scientific investigation of objective values, but perhaps only in the distant future. Still, it is interesting to consider that the frontiers of rigorous scientific understanding might extend beyond the limits of the principle of causality, even in making sense of mental processes.

Intentionality has occupied a central place in the science of psychology as a central concept of folk psychology rather than as a valid scientific principle. It is invoked in how people understand each other and themselves. Separately, there has been interest in folk physics: how people understand the physical world. People use implicit versions of the principles of intentionality and causality for their integrated mental models of the world, making them models of What-Matters. In a similar way, the scientific intentional model outlined below is a What-Matters model that fully employs the principle of causality, not only as to physical change, but as an influence on the development of intentions, and a common distorting influence on an agent’s baseline intentions. Neuroscience will continue to advance in predicting human behavior by means of wholly causal (What-Is) models. Intentional models of the mind will fully incorporate those causal influences, both as alternative explanations of baseline intentional influences, and as explanations of how baseline intentions get distorted. It is reasonable to suspect that models of the mind and models of the brain will tend toward identical predictions of human behavior, while offering dramatically different explanations.

## The Dennettian Model

The general structure for intentional models is fairly familiar. It has been stated with exceptional clarity in Dennett (1987). He proposed it as a description of folk psychology, but suggested that it is so powerful that it could be the basis of a scientific model of intentionality, saying that it “seems to be a *true* theory, by and large, and hence is a candidate… for incorporation into science” (p. 47). This article follows and elaborates on his proposal, except that a method is proposed for tracing temporarily irrational behavior to identifiable causal influences.

According to Dennett, the heart of folk psychology is the taking of an intentional stance as the primary way to predict human behavior, as well as the behavior of various other complex systems. This stance treats people as rational agents who choose in conformance to their beliefs and desires. Thus, it is possible to assess what the agent *ought* to do, and then infer what beliefs and desires they must have in order to get to that rational behavior. Beliefs, then, are invented constructs of the theorizer, rather than actual entities.

In order to predict behavior, he says (p. 17), “you figure out what beliefs [and desires] that agent ought to have, given its place in the world and its purpose.” Then you figure out what “the agent *ought* to do” in this situation (what the agent will do if rational), and that is the behavior you predict. He actually suggests a pragmatic approach to modeling desires: start with the most basic, such as the desire to survive, eat, procreate, find entertainment, and avoid pain, plus desiring to do other things as the means toward those ends. He says that one must develop “special stories” to account for an agent’s false beliefs and detrimental desires that result in irrational behavior (p. 20). He is referring to stable irrationality, whereas the theory below accounts for variable rationality. Dennett calls the intentional stance “an extraordinarily powerful tool in prediction” (p. 24) until the area to be predicted gets too fine-grained. The Dennettian model mostly treats agents as consistent, guided by stable beliefs and desires. The intentional model proposed below attributes inconsistencies in an agent’s behavior to causal influences, and suggests that it can be refined to produce accurate predictions even at fine-grained levels.

Dennett acknowledges that it seems circular to attribute beliefs and desires to an agent by assuming that she is acting rationally, and yet to determine what would be rational for this agent based on what she ought to do *given* her beliefs and desires. But the “whole system of interlocking attributions… is saved from vacuity by yielding independently testable predictions” (p. 50). In much the same vein, this article suggests that the science of psychology has implicitly employed the folk psychology model as a starting point, and has developed methods for making and testing such predictions.

Dennett takes pains to distinguish the brain as a “syntactic engine” (a kind of organic computer) from the mind as a semantic engine, operating more by meanings [and purposes] and their complex interconnections than by automatic procedures. He says that “individual beliefs and desires are not attributable in isolation, independently of other belief and desire attributions” (p. 58). It is necessary, then, to understand the whole mental model. It is of interest how a semantic engine is realized by a syntactic engine, but there might not be a causal relation between the two, because “the syntax of a system doesn’t determine its semantics” (p. 61). The implication is that brain science investigates behavior based on syntactical structures, whereas mind science investigates by semantic structures: meanings with belief and desire aspects. The explanatory path for brains is in causal chains through neural pathways and into the past, whereas the explanatory path for minds is outward into the environment and forward into the desired future.

Dennett denies that there is a fixed point of distinction between being seen as a syntactic or a semantic engine (e.g., pp. 31–32). But at some point of complexity, the semantic interpretation is the more powerful. In particular, if the system (such as a person) seems to have an internal representation (a mental model) that sufficiently fits the environment, it is treated as an agent. His point is that science builds models of minds by recognizing patterns that are in some sense real, apart from causal patterns in the brain. Again, the intentional model is built around constructs such as belief. According to Dennett, such constructs are more real than instrumentalist, but they might not have determinate content (pp. 39–41).

## The Intentional Relation

Brentano (1874/1973) proposed that an intentional relation is an aboutness relation between a meaning and whatever it is about (whatever it points at). For the proposed scientific model, however, the intentional relation is recast as “I intend it,” an I-it (subject-object) relation that is mediated by the mental meaning by which the subject points at (characterizes and values) the object.^5^ In a belief, the subject implicitly asserts that this meaning accurately represents that state of affairs, past, present, or potential. In a perception (which is a sort of belief in this sense), the pointing is spatial, such as the subject using her *apple* meaning to identify that object. In a desire, the subject is attracted to or repulsed by an imagined future that is characterized by beliefs.^6^ In its belief aspect, intention is mere aboutness. In its desire aspect, it is also an influence on overt or covert behavior.

Desire, as used here, is any affect/feeling (such as urge, mood, or emotion) that influences choice or value judgment. Zajonc (1980) suggests that “the form of experience that we came to call feeling accompanies *all* cognitions…” (p. 154), where “affect and cognition are under the control of separate and partially independent systems that can influence each other…” (p. 151). He distinguishes approach/avoidance feelings from other sorts of feeling like surprise and guilt (p. 152), thus seeming to distinguish intentional affect from other sorts of affect. Biologist Freeman (2000) says, “All actions are emotional, and at the same time they have their reasons and explanations. This is the nature of intentional behavior” (p. 210). Consistent with this claim, the intentional model treats each intention as having both a belief and a desire component.^7^ Beliefs model what is so and what is likely. Desire (affect that influences choice or value judgment) is implicated in both the direction and intensity of tendency to act.

The subject in the intentional relation is something like a content-free, merely implied “I”, with all the content (such as a self-concept and a means of customizing desires to each situation) contained in the mental model. Actions are guided by what the subject (the agent) intends to accomplish, given what she understands of her interests, the situation, and the likely consequences of available choices that come to mind. Thus, the subject is in charge, but there is no apparent conflict between this intentional model and a causal model of the brain, because both the choice of action and the degree of motivation are modeled as entirely reflecting the mental state by which the subject intends, which is presumably underlain by a knowable brain state.

The intentional relation (I intend it) is independent of the causal relation (A caused B), such that it is unlikely that the one can be derived from the other. Whereas the causal relation suggests a model structured around the laws of nature, the intentional relation suggests a structure that can be characterized as the personal perspective of a subject (agent). The perspective implies an entire mental model that is the lens by which the subject’s environment can be brought into focus. That is, the subject’s mental model provides an implicit context for all experience, and primed beliefs and desires (such as recent perceptions) provide a somewhat less implicit model of the environment as the immediate context. The specific intention occurs within this context. Intentions, including perception, are necessarily attributed to an agent using such a lens. In order to predict and explain human thought and behavior intentionally, the science of psychology develops a scientific model of the agent’s mental model. Rather than treating it as accessing fixed or definable beliefs and interests (things that are desirable), the mental model is more appropriately treated as a tool for customizing beliefs and desires to the situation. Even in a highly unfamiliar situation, this mental model tends to make associations to meanings that might apply. Whereas causal models of human thought make a rather sharp distinction between the cerebral cortex as the source of beliefs and the limbic system as the source of desires, an intentional model tends to treat of whole meanings, their interrelations, and the broader context.^8^

It is noteworthy that Brentano discussed the intentional relation in a book called *Psychology from an Empirical Standpoint.* According to Bartok (2005), he was especially interested in proposing a scientific (empirical) methodology, quite apart from the philosophy, although the empiricism he was thinking of was phenomenological. The proposed intentional model, in contrast to Searle (1983)^9^ and perhaps Brentano^10^, treats all meanings as intentional (as characterizing and valuing the intended object, even if not currently motivating action). Meanings in a fantasy are about imaginary objects, and one’s *apple* meaning during a stream of thought, even if a scientific consideration of the class of apples, has evaluation as one semantic dimension.^11^ Perhaps most or all voluntary mental processing is intentional in this sense, supported by involuntary processes such as memory search and predictive coding.

## Wisdom And The Principle Of Intentionality

Two centerpieces of the proposed intentional model remain to be specified: the construct of objective wisdom and a formal statement of the principle of intentionality.

Wisdom, as used here, is a measure of the practical understanding and rationality of intentional beings (of believing what you ought to believe and wanting what you ought to want, as Dennett put it; see also Baltes and Smith, 1990). Wisdom is an objective standard for what Dennett calls rationality. That is, rational behavior is doing what is rational according to your beliefs and desires, and wisdom is a measure of the adequacy of those beliefs and desires. Roughly, intentions are wise to the degree that they are likely to bring about desirable situations and desirable lives, with individual differences in the wise rate by which delayed and longer-term benefits are discounted. Wisdom is a measure of the conformity of a subject’s beliefs to what is so, and of her desires to what is prudentially valuable to her. Prudential value is what that subject *would* desire if she were wiser, and thus reflects what is so about what is desirable to her in net.^12^ Discovering what is objectively wise is sometimes a goal of psychology (e.g., Greene and Brown, 2009). Some psychological research chooses situations in which the normative (objectively wise, according to society) behavior is known, such as a correct judgment.

Intentionality has a practical function in life just because some intentions are objectively more adequate (wiser, more rational, and more adaptive) than others.^13^ Although wisdom might be inherently rather fuzzy, it can be made increasingly objective as a measure, especially in controlled contexts. As Dennett points out, intentions are inferred from what is wise, and wisdom is inferred from what is intended, anchored by independently testable predictions such as occur in psychological research. Wisdom deficit of two sorts is important to an intentional model. Ignorance is the deficit in a subject’s baseline wisdom when compared to objective wisdom, whether that deficit is due to lack of knowledge, a misunderstanding of actual interests, or a defective reasoning process. Foolishness is the further deficit in a subject’s temporarily distorted wisdom when compared to her baseline wisdom.^14^

The constructs of objective wisdom, ignorance, and foolishness suggest a framework for the prediction and explanation of intentional behavior around the intentional relation and the notion of mental models. The proposed principle of intentionality might be stated as follows:

Every intentional act is guided by what is designated as wise by the subject’s currently active mental model, whether it is the subject’s baseline model (measured for adequacy against objective wisdom) or a deviation therefrom (foolishness) that is traceable to distorting influences on the subject’s beliefs and desires.

This principle models intentional behavior around three levels of wisdom: objective, baseline, and foolish.^15^ Consistent with Dennett, the notion of objective wisdom is necessary in order to provide an anchor point around which intentionality can be tied to what is so. Still, to be intentional is to pursue whatever *seems* wise, based on the currently active mental model. A depressed subject, for example, models the world abnormally, and behaves intentionally (wisely) based on that distorted (foolish) model. Equally, the beliefs and desires of an ongoing depressed state might at some point be treated as the new baseline.

A subject’s casual choices are sometimes inconsistent with her baseline mental model, even in the absence of distorting influences. It is reasonable to assume that only a tiny (and not always very representative) slice of the subject’s baseline model is accessed for casual choices. A subject might rely excessively on salient and primed factors in a judgment (see, for example, Taylor and Fiske, 1978), or fail to take pertinent beliefs and desires into account. Intentional behavior is conceived as wise, *based on* that tiny slice. Thus, some foolishness is attributable to accidental failure to take key considerations into account, traceable to involuntary processes that determine which beliefs and desires get activated. The power to predict and explain casual behavior intentionally is dependent on modeling those processes. Further, in very fine-grained prediction, the principle of intentionality is most effective when operating alongside functional models of the brain. The study of intentionality is, for now, most effective in controlled, well-designed situations.

This intentional model is most obviously applicable to behavior in pursuit of one’s intentions. But by hypothesis, each meaning activated during a thought processes has belief and desire aspects, so that it might subtly influence the direction of thought. There are probably also involuntary cognitive processes that have little to do with belief or desire, and yet influence intentional behavior. For example, if an agent intends to place a bet on the correct roulette number, processes other than intentionality might influence what number is chosen. As another example, well-practiced, rule-based processes such as memory search surely interact with intentional processes in some mental processing.

The intentional model is particularly apt for describing those occasions in which top-down processes guide choices and behavior. The desired future can sometimes be modeled in a nesting of levels.^16^ When a domino falls, a scientific explanation might trace it backward in space-time through a row of dominoes to the finger that pushed the first one. Instead of continuing that causal chain through functional processes and influential external events, the explanation might instead continue in a nesting of desires, where a grandmother started the domino chain reaction to entertain her grandson, to enjoy his reaction and encourage his liking of her, to build the bond between them, to enhance the desirability of her life. Such nesting, although implicit and not always conscious, is subject to empirical testing.

Notice that this principle of intentionality, when combined with neuroscience and cognitive modeling, might someday be adequate to predict and explain all voluntary behavior, even if fine-grained, in ways that are consistent with ordinary notions about mind.

## The Intentional Model And Mainstream Psychology

Dennett suggested that the intentional stance cannot be successful in fine-grained prediction of behavior. But a great deal of psychological research over the past hundred years has implicitly used something like the Dennettian model, and has been successful in developing techniques that make it increasingly manageable. Individual differences are almost eliminated by modeling the intentions of the *average* subject. Extraneous influences are virtually eliminated by experimenting under controlled conditions. And researchers carefully design situations that isolate some narrow aspect of the average subject’s beliefs and desires, mapping the influences on behavior by incremental changes in the independent variable. The scientific model of the mind of the average college freshman is extended one narrow research area at a time.

Besides exploring the rational behavior that is guided by the subject’s baseline beliefs and desires, some psychological research introduces variables that are designed to distort the subject’s baseline desires (or, sometimes, baseline beliefs). Notions like wisdom, ignorance, and foolishness are implicit in any psychological research that compares control group behavior both to normative behavior and to the deviant behavior of experimental groups. Models of irrational behavior are facilitated by the assumption that any deviation from baseline behavior indicates a temporary distortion of the subject’s baseline desires and/or beliefs, where that distortion is attributed to involuntary brain processes that can often be further traced to external influences such as the independent variable. In a typical experiment, a control group defines baseline behavior, and experimental groups are exposed to independent variables that are expected to trigger something like a limbic system activation such as a feeling of greed, anger, self-doubt, or fear. In some research, the independent variable is expected to distort beliefs or judgments, such as by priming a meaning that might interfere.

Baseline intentions might usually be designated as what is normal for that subject. But ‘normal’ is a bit ambiguous. For example, Kahneman (2011) describes System 1 and System 2 judgment processing, in which System 2 judgments are more careful and effortful. As he notes, it is rational to do System 1 judgment processing when the stakes are low, and System 2 processing when they are sufficiently high. Either one, then, might be treated as baseline intentions, depending on the purposes of the research.

One of the merits of the proposed intentional model is that it facilitates mixing causal and intentional influences in whatever ways are convenient. Presumably, all behavior is underlain by neural processes, so that the researcher is free to specify which aspects of behavior are to be modeled intentionally. There might, for example, be aspects of voluntary behavior that are more conveniently modeled as functional brain processes for a particular research program.

There are powerful functional descriptions of neural processes that tend to isolate how the brain mimics intentions. Functional models, then, might provide a valuable interface between models of mind and brain. Dennett suggests that the intentional stance works because “evolution has designed human beings to be rational, to believe what they ought to believe, and to want what they ought to want (p. 33).^17^ He goes on to say (p. 34) that “a currently… popular explanation is that the account of how the [intentional] strategy works and the account of how the mechanism [the brain] works will (roughly) coincide… I think some version of [this explanation] will prove correct.”

The principle of intentionality empowers the prediction and explanation of human behavior based on beliefs and desires, and provides the structure for a complete intentional model. That model has practical limitations in the near-term, just because the construct of objective wisdom needs fleshing out. The most obvious value of the principle is in controlled situations in which it is possible to define an operational construct of wisdom. Beyond that, the principle of intentionality might have immediate application in inspiring novel hypotheses for explaining intentional behavior, and in suggesting synergies between various existing psychological theories. It might also have immediate application in any area in which it is useful to make attributions to both intentional and causal influences, such as psychophysical investigations (signal detection, context effects, etc.) and research on extended processing, where it might facilitate the combination of rule-based and intentional aspects. And it has obvious application in learning and developmental models, where the concept of objective wisdom can be valuable.

## Explaining Intentions Within Wholly Causal Models

Some models of cognitive processing, such as connectionism (e.g., Rumelhart and McClelland, 1987) and predictive coding (summarized in Clark, 2013) are highly successful without addressing intentionality head-on. It seems likely that they will continue to predict immediate interactions with the environment in finer-grained detail than can be accomplished by any model based on the principle of intentionality in isolation from these functional models. But models of human cognition as internal information processing are sometimes criticized for treating the brain as if it were a computer processor, and in some ways a black box. This has led to alternative, functional approaches that extend beyond the brain to body and/or environment, typically taking the phenomenology of Merleau-Ponty (1962) as a starting point. Examples include embodied cognition (Rosch et al., 1991), situated cognition (Clancey, 1993), enactivism (Thompson, 2007), and externalism (Clark and Chalmers, 1998). Some of these approaches employ intentional language in describing processes that are finally causal. Weber and Varela (2002) think that it is a mistake to ignore the fundamentally teleological nature of life. Portraying the viewpoint that they oppose, they say, “In our present scientific world…the teleological behavior of living beings is an illusion, an appearance hiding the underlying mechanism” (p. 100). Instead, they suggest, “organisms are subjects having purposes according to values encountered in the making of their living” (p. 102). Di Paolo (2005), following Varela (1991), proposes that anything is an agent that has adaptive autopoiesis, even at the level of a single-celled organism. He calls such an agent “a self-constructed unity that engages the world by actively regulating its exchanges with it for adaptive purposes that are meant to serve its continued viability” (p. 443). Thompson (2011) proposes that “advances in biology and the sciences of mind and brain can properly address issues about the teleology of life and the intentionality of consciousness” (pp. 10–11). Thompson and Stapleton (2009) criticize “the traditional functionalist conception of cognition as fundamentally distinct from emotion” (p. 27). As they put it, “neurons do not think and feel; people and animals do” (p. 26). But taking the influence of affect into account does not, by itself, speak to whether enactivism is a form of teleological functionalism. Rowlands (2009) says that enactivism “seems to be a specific form that functionalism might take” (p. 57). All of these approaches account for future-directed behavior without coloring outside the lines of a wholly causal model. They are consistent with the idea that intentional mental processes are emergent from brain processes in ways that, although finally causal, do not fit easily into the classic version of causality as a unidirectional chain of causes.^18^

But all functional processes, even if they extend beyond the brain and include affect, are part of what can be seen as causal modeling, and thus as value-neutral. Nagel (1977), in discussing biological teleology, makes a key distinction between intentional and functional teleology. Here is how he describes the “intentional view.” The “goal *G* of an action or process is said to be some state of affairs *intended* by a human agent; the *intention* itself is an ‘internal mental state’ which, coupled with the internal state of ‘wanting’ *G* together with ‘believing’ that an action *A* would contribute to the realization of *G*, is allegedly a causal determinant of the ensuing action *A*” (p. 264, emphasis his). Uses of intentional terms such as agent, purpose, and value in functional teleology are what Nagel calls a ‘metaphorical extension’ beyond the intentional view (p. 266).

On this topic, Dennett offers a telling commentary: “But the brain… is just a syntactic engine… That’s all brains *can* do… How could *any* entity… get the semantics of a system from nothing but its syntax? It couldn’t.” (p. 61). He goes on to say that the brain simply mimics semantics. For the science of psychology, this suggests that the intentional model of the mind is independent of the causal model of the brain. There is considerable value in identifying processes that mimic intentionality, but it misses the heart of what it means to be intentional.

Consistent with what Nagel calls the intentional view, the proposed intentional model assumes that subjects freely act in pursuit of whatever they find desirable at the moment, given their active beliefs and what they expect to be the effects of available actions. Intentions have content and connection to the world based on objective measures of their adequacy. A belief can be compared to what is so, and a desire to what that subject would have wanted if she had better understanding of herself, and used better judgment processes. The more objectively adequate an intention the more it tends to be rewarded, which is evidence of what is objectively wise. Although Dennett and Nagel are very far apart on the topic of consciousness, and perhaps as to the *causal* efficacy of intentions, there is no apparent conflict between them as to the nature of a scientific model of intentionality.

## Putting The Intentional Model Into Context

Various approaches to intentional modeling have been attacked. For example, Carruthers (2013) denies that judgments and decisions are guided by concrete, introspectable intentions (such as inner speech). But he is simply insisting people infer their own intentions in much the way they infer the intentions of others. Elsewhere he supports intentions as being efficacious. For example Carruthers (2008) argues that minds “are organized into sets of perceptual systems which feed into belief-generating and goal-generating systems, and which also inform practical reasoning in light of the goals so generated” (p. 260). As with the Dennettian model, this treats beliefs as constructs inferred scientifically, rather than necessarily being determinate, introspectively available, or even proposition-like.

Gauker (2005) asserts that philosophy has been unsuccessful in finding any law-like formula for predicting rational intentional behavior. “People do what they believe will satisfy their desires” is an example of the sort of formula that he criticizes (p. 122). He does not, however, deny that intentions are efficacious. Instead, he is saying “that we cannot conceive of the rationality of action as conformity to some all-purpose rule” (p. 142). The proposed principle of intentionality is a law-like model, and should be powerful even now in various arenas. However, the power of this model in predicting behavior will increase only as all sorts of blanks are filled in by empirical data and new theoretical structures, dealing with complexities such as those described above.

This article has suggested that behavior can be broken into two very different models. Brain science is based on the principle of causality, whereas mind science ought to be based primarily on the principle of intentionality and only secondarily on causality. The intentional model will always be at least as powerful as any wholly causal model, because it fully incorporates causality in whatever ways make intentional predictions most accurate.

Whereas a causal model traces behavior to external and past influences, an intentional model traces it to interpretations of the environment and to the imagined desirable future (as well as to distorting causal influences on the subject’s baseline beliefs and desires). These seem to be two different *methods* of explanation, rather than competing claims. One method traces all consistencies through the relation “A caused B,” with the other adding “I intend it” as an alternative. There are advantages to retaining two models, where the mind is a valid construct in one model but not the other; where there are subjects with free will in one model but not the other; and where it is bad to starve to death for lack of food in one model, leaving the other model value-neutral. The principle of intentionality is presumably the more appropriate approach to giving content to constructs such as mind, meaning, belief, desire, “I”, purpose, happiness, and value.

The principle of causality treats causes as controlling what happens. The principle of intentionality treats intentions as controlling what happens, not causally, but by the agent’s power to act. The notion of free will has to do with an agent controlling as a sort of first cause, rather than being modeled as a link in a chain of causes.

## Modeling What-Matters

It is surely no accident that Dennett says that the science of intentionality predicts based on what the agent *ought* to do. Any wholly causal model is simply descriptive, and can only bring value into the discussion with IF/THEN statements, such as IF you want scientific progress, THEN it is valuable to…). By contrast, any intentional model revolves around discovering and pursuing what is desirable. It is inherently prescriptive in addition to being descriptive, in that it prescribes behaving rationally and pursuing wisdom, by developing truer beliefs and wiser desires: intentions that align ever better with bringing about overall personal good.^19^

There is significant scientific understanding of practical truth, but greatly limited scientific understanding of personal good. Thus, a scientific model of What-Matters personally (beyond what is wise in carefully controlled situations), is currently little more than a dream. Eventually, models of value might gain traction, as models of mental processes become more advanced. All of this is reminiscent of the earlier Dennett quote about rationality, where independently testable predictions and interlocking attributions can gradually help to produce an internally consistent model of mattering.

The intentional model is as applicable to a community as to individuals – to a community of minds in addition to an individual mind. But perhaps the common good is even less accessible to rigorous scientific investigation than is the personal good of the average subject.

## Implications For Philosophy Of Mind

The intentional model highlights a distinction between three kinds of knowledge. First, there is objective knowledge about physical objects such as the brain. It is objective in two ways: being about physical objects and being intersubjective, scientific understanding. In addition to physical objects, it includes objective (intersubjective) knowledge of scientific constructs such as space-time, energy, and causality, whatever their ontological status. Second, there is objective knowledge about people and other physical objects that employs the intentional model. It, too, is objective in both ways, and it adds objective (intersubjective) knowledge of scientific constructs such as subject, mind, purpose, value, and wisdom, whatever their ontological status. This surely counts as objective knowledge, even though knowledge of mental processes is currently less advanced than knowledge of the physical. Third, there is *subjective* knowledge about phenomenological experiences like pain, such that I know how today’s pain differs from yesterday’s pain. It is subjective in that, when I describe the difference to you, you can only infer what I mean by assuming that we have similar phenomenological experiences in objectively similar situations. The subject is an objective construct of the intentional model, but the experience of *being* a subject is phenomenological, something like what it is like to be a bat (Nagel, 1974). The causal model of the brain and the intentional model of the mind have in common that they are objective models, with the intentional model employing constructs missing from the causal model. These two models serve different purposes and use different methods of explanation, so that the intentional model might never be adequately subsumed within a wholly causal model, even if intentional knowledge finally adds nothing to predictive power as to behavior.

Thus, the science of intentionality does not address phenomenological issues such as the nature of qualia. McGinn (1989) denies that there can be any final solution to the mind-body problem, because you can only investigate by studying brains or introspecting. This argument seems only to address the causal model versus phenomenology. This article has suggested that a different mind-body problem (leaving phenomenology aside) is solved by recognizing that there are two valid scientific models that serve different purposes.

Philosophers of mind such as Davidson (1970) theorize how mental events can be causally efficacious. This article has suggested that, within the wholly causal model, mental events are non-existent, and thus neither causally efficacious nor epiphenomenal, as far as science is concerned.^20^ Instead, they are intentionally efficacious, personally guiding behavior. Pain, for example, can be intentionally efficacious, apart from the phenomenology, based on the meaning given to it, with both perceptual and desire components. Bem (2001) promotes explanatory pluralism in a way that seems to make room for the proposed intentional model. He describes the psychological level of explanation as functional but not causal, by accepting Brentano’s notion of aboutness as a valid functional explanation rather than a metaphysical concept (p. 789). Robinson (2010), a philosopher trained in psychology, says, “Mental life is a life of meanings. Physical entities are quite literally meaningless. I take that gap to be unbridgeable” (p. 791). That seems consistent with the principle of intentionality, regardless of the ontology of intentionality, and quite apart from phenomenology.

Dennett’s point with the intentional stance is that it is an invented model that works, rather than mirroring the structure of reality. The scientific value of the proposed intentional model is independent of the explanation for why it is so powerful. Still, it is striking that there might be no appropriate way to fold the intentional model into any wholly causal model. It is possible, then, that there are two valid models of the universe that cannot be combined. There are at least three approaches to treating both as valid.

The first is simply pragmatic: powerful but conflicting theories are sometimes both treated as valid, until one is undermined or subsumed under the other, or the two are subsumed under a unifying theory. This is the case with the coexistence of quantum theory and relativity theory. Both theories make powerful and almost identical predictions of gravitational effects, but with very different explanations. Each has ongoing value in somewhat different arenas, but there is some expectation that the two will eventually be rolled up into a unified theory that is superior to either theory alone. In the same way, it might turn out that the What-Matters model can be wholly subsumed within the dominant scientific (causal) view of the universe, or that a new, unifying model will arise.

A second approach is under-determination of the sort that Quine (1975) proposes, such that two (or more) empirically equivalent scientific models survive indefinitely, with no scientific way to choose between them. There might always be one model based on the principle of causality and a second model that also includes the principle of intentionality, with no scientific way to choose between them. Because these two models have very different metaphysical implications, there might be no way to resolve the disagreement between believers and disbelievers in genuine agency. Those who deny that the principle of intentionality mirrors anything in ultimate reality might continue to rely on the intentional model in their everyday lives. That is, people who reject the notion that there is agency in ultimate reality can still warmly embrace intentionality as central, both in their own personal lives and as a model with unlimited value in prediction and explanation. This might be a good representation of Dennett’s view. It is also possible that neither the principle of causality nor the principle of intentionality mirror anything in ultimate reality. It is possible, for example, to adopt a Kantian view that both are merely *a priori* categories of the mind.

The third approach assumes that the structure of reality is more complex than can be represented in any wholly causal model. Think of ultimate reality as having two interconnected dimensions, one causal and the other intentional. The intentional relation is real, where the intentions of an agent actually influence physical events, and where the agent is free to change her mind. But, by hypothesis, there might be no way to confirm this direct influence experimentally, because the intentional and causal dimensions are inextricably connected and fully in harmony. Neural processes and external influences that can be traced in causal chains into the past are integral in the formation of intentions, such that, in principle, a wholly causal model could powerfully predict what the agent will intend. The agent’s intentions directly influence physical events, but there is an epiphenomenal causal link alongside that influence, so that there is no scientific basis for choosing between the two explanations. It is, of course, conceivable that there will eventually be some experimental means that we cannot yet imagine for sorting out whether it is actually the intention that is efficacious. A two-dimensioned universe of this sort is independent of the dualistic claim that conscious experiences (qualia) are real but non-physical. A dualist cites the evidence of subjective experience to justify the reality of qualia. But a proponent of the two-dimensioned universe might, instead, cite the utter inadequacy of any wholly causal model for making sense of anything about minds and mattering. And it might further be argued, consistent with the dualist argument, that the experience of free will and things mattering is more primitive and certain than any causal model of the environment.

## Author Contributions

The author confirms being the sole contributor of this work and approved it for publication.

## Conflict of Interest Statement

The author declares that the research was conducted in the absence of any commercial or financial relationships that could be construed as a potential conflict of interest.

## Footnotes

1 Einstein denies that even firmly established principles like *E* = *mc*^2^ are anything more than theories: “Physical concepts are free creations of the human mind, and are not, however, it may seem, uniquely determined by the external world.” *The Evolution of Physics*, A. Einstein and L. Infeld, New York, Simon and Schuster, 1938.

2 There are unexplained trends for which no causal explanation is offered, but perhaps no claims that such trends are independent of complex causal explanation.

3 The notion of cause is not without controversy, such as Russell (1913) and Norton (2003).

4 Hume (1739/1978) famously concluded that there seems to be no way to know whether causal efficacy is real, beyond inference from experience.

5 Kriegel (2016) argues that Brentano ended up thinking of intentionality as a property of the subject rather than the meaning, although not quite as is suggested here.

6 Describing a desire in propositional language, it might be called the assertion that such-and-such future state would be beneficial or satisfying. Such an assertion brings rationality (and thus an objective standard) to bear on desires.

7 Beliefs and desires might be treated as mere constructs, segregating an intention artificially into contributions from cognitive (representational) and affective (motivational) processes. The desire component of a meaning might sometimes assign a neutral value, implicitly asserting that its object is neither attractive nor repulsive in net.

8 The belief portion of a mental meaning, for example, might sometimes draw part of its content from beyond the cerebral cortex. Freeman (2000), speaking of the map-like contributions of the hippocampus says, “Intentional action cannot exist without this learned framework, but it is a dynamic operator, not a repository of facts or geometric forms.” (p. 217)

9 Searle (1983) says, “Some, not all mental states and events have Intentionality. Beliefs, fears, hopes and desires are Intentional; but there are forms of nervousness, elation and undirected anxiety that are not Intentional.” (p. 1) In the proposed intentional model, however, it is possible that such affective states are not mental states until given meaning (where *I am anxious* is a belief and being anxious is undesirable), so that it does not conflict with Searle.

10 Bartok (2005) says that by the time of the 1911 appendix to his book, Brentano had more deeply considered the ontological issues, and did not think that there could be an intentional relation to a non-existent object (p. 22). Whether or not Brentano was right, the proposed intentional model addresses scientific rather than ontological intentionality.

11 Osgood et al. (1957) found that evaluation such as attraction and repulsion was the largest single dimension of most meanings.

12 Prudential value is a term that philosophers (e.g., Griffin, 1986; Taylor, 2013) sometimes employ for something like what I am calling wisdom. It is reflected in later regret and in patterns of learning from experience.

13 The adequacy of intentions is often measured against cultural norms, the adequacy of which must finally be measured against objective wisdom, even though objective wisdom might vary a bit from one culture to another, just as it does from one person to another.

14 People whose baseline wisdom is worse than the norm of a culture are sometimes called foolish. In the proposed terminology they would instead be called ignorant. However, a subject who consistently takes action she knows to be foolish, ignoring the likely consequences, is modeled as behaving foolishly, with her baseline wisdom repeatedly distorted by limbic activations.

15 Better-than-baseline rationality or greater-than-baseline enthusiasm might be considered a normal (rational) response to exceptional conditions. It would only be foolish if, according to that subject’s baseline wisdom, the effort were inappropriate to the potential outcome.

16 Bratman (1987) discusses this sort of nesting of intentions as pieces of larger plans.

17 Rosenberg (1994) argues that evolution selects for function rather than structure, biologically. From the perspective of the proposed intentional model, an assertion like Dennett’s might mean that evolution can be seen to select for rationality more than for neural structures. This lends support to the claim that there are objective measures of the adequacy of intentions.

18 See, for example Cabell and Valsiner (2014).

19 People are social animals, so that personal good is inseparable from the welfare of others, but this discussion will ignore that complication.

20 As quoted above, Nagel (1977, p. 264) is among philosophers who treat the intentional view as alleging that intentions are *causally* efficacious ontologically.
